# Understanding health literacy within the nexus of environmental, planetary, and one health: Mapping the evidence via bibliometric content analysis

**DOI:** 10.1016/j.joclim.2026.100700

**Published:** 2026-06-26

**Authors:** Lydia Reismann, Jennifer Kallenbach, Carmen Jochem

**Affiliations:** a Chair of Planetary & Public Health, University of Bayreuth, Universitätsstraße 30, 95447 Bayreuth, Germany; b Faculty of Human Sciences, Department of Education, Medical School Hamburg, Am Kaiserkai 1, 20457 Hamburg, Germany

**Keywords:** Public health, Environment, Climate change, Sustainability, Health education, Animal health

## Abstract

**Background:**

Amidst an anthropogenic climate and ecological crisis, the conventional understanding of Health Literacy has been expanded to address socio-ecological determinants of health through the Planetary Health Literacy, Climate and Health Literacy, or One Health Literacy concepts. However, the field's interdisciplinary character and its recent evolutionary status have led to theoretical fragmentation and a lack of conceptual debate.

**Methodology:**

To test three expectations regarding the conceptual underpinnings of literacy and competency concepts and to systematically map the evidence, a bibliometric content analysis is conducted using co-occurrence analysis (COA).

**Findings:**

A total of 135 peer-reviewed studies meeting the inclusion criteria are analyzed from 1444 screened publications. COA via bibliometric network viewer VOSviewer yields the formation of four distinct clusters: 1) Competencies in Planetary Health Education and sustainable healthcare; 2) Environmental Health Literacy and environmental risk and exposure awareness; 3) Competencies regarding One Health, the animal-human-health nexus and interdisciplinary education; 4) Food Literacy, nutrition, and sustainability. Analyzing patterns of single items underscored One Health and Planetary Health as broadly integrative themes, whereas other concepts remained cluster-specific. The hypothesized distinction between One Health and Planetary Health Literacy is omitted, whereas conceptual differences between Environmental Health Literacy and Planetary Health Literacy are confirmed.

**Conclusion:**

The findings contribute to theoretical and methodological research trajectories and reveal the widespread use of vague terms such as *knowledge, skill,* or *attitude* over differentiated literacy constructs. The results underscore the need for standardized terminology and conceptual underpinnings, particularly within educational research.

## Graphical abstract

**Figure:**
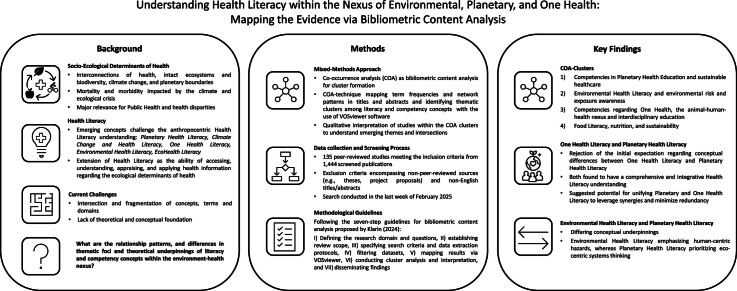

## Introduction

In the past decade, the Public and Global Health discourse has shifted towards understanding upstream determinants of health, including systemic, structural and ecological drivers that influence individual and population health outcomes [[1], [2], [3]]. Key among these are planetary boundaries, such as a stable climate and intact ecosystems, which are recognized as foundational to sustaining human and animal health [4,5].

Prior research reveals a stark disconnect: while anthropogenic climate and ecosystem changes are widely perceived as major crises, their profound health implications remain poorly understood by patients and inadequately addressed in health profession education [[6], [7], [8]]. Health professionals often lack self-assessed knowledge and literacy in climate-health linkages, limiting their capacity to drive transformative action [6]. Concurrently, evolving concepts such as Planetary Health Literacy (PHL) [9], Climate and Health Literacy [10], One Health Literacy (OHL) [11], EcoHealth Literacy [12] and other comprehensive Health Literacy concepts [7] have sought to bridge this gap and have synergistically contributed to a broadened understanding of Health Literacy by integrating and addressing the distal and ecological determinants of health. For example, the recent development of the definition of PHL as “the knowledge and competencies of accessing, understanding, appraising, and applying information in order to make judgements and take decisions regarding planetary health, across societies and for health-promoting, sustainable, and transformative actions” [9] has been influenced by the other concepts mentioned above. In this manuscript, we refer to disciplines such as Environmental Health (EH), One Health (OH), or Planetary Health (PH) as the underlying approaches, and their related literacy/competency constructs as literacy concepts. Their scope extends beyond health profession education, for example envisioned by the German Advisory Council on Global Change recommendations, representing a lifelong learning approach to integrate planetary health-related literacy and competency across formal and non-formal education [13].

Despite the overlap between the different approaches, the field remains fragmented, lacking a cohesive theoretical foundation or standardized taxonomy to differentiate these intersecting domains which leads to scientific debates [[14], [15], [16]]. We acknowledge that "competency" and "literacy" are used differently across disciplines, such as in formal education, health literacy research, and public health practice, often referring to distinct yet overlapping combinations of knowledge, skills, and contextual application. Given the absence of a consensus definition and inconsistent usage in the analyzed approaches (PH, OH, EH), it remains unclear which conceptualization underpins each. This review adopts an understanding of "competencies" as a developed set encompassing the necessary knowledge and practical skills for transformative action and decision-making, while "literacy" is used to refer to a specific concept, in particular incorporating specific health literacy domains.

As part of our ongoing research, we conducted a systematic scoping review to synthesize existing literature and understand the definitions of key concepts within the environment-health-nexus [17]. However, its methodology was insufficient to disentangle the temporal shifts and nuanced distinctions of concepts or contextualize their co-evolution within broader disciplinary approaches. For a better understanding of the conceptual landscape of literacy (L)/competency concepts tied to the environment-health-nexus—specifically EHL, OHL, PHL—we adopted a bibliometric content analysis guided by the overarching research question: What are the relationship patterns, and differences in thematic foci and theoretical underpinnings of literacy and competency concepts within the environment-health nexus? To our knowledge, this represents the first effort of its kind to address this research question.

Drawing on unpublished data from an ongoing systematic scoping review [17] and prior literature search, we posit three expectations that are the subject of this study.

### Expectation I) divergent scopes of OHL and PHL

We expect that if the approaches PH and OH differ in their disciplinary scopes, their associated literacy concepts (PHL and OHL) will reflect these distinctions. Historically, OH has emerged from veterinary and disease ecology research fields, prioritizing zoonoses and antimicrobial resistance, whereas PH has adopted a holistic, systems-oriented perspective emphasizing interconnected planetary boundaries [14,16]. Despite the recent conceptual broadening of OH, its research remains anchored in animal-human health interactions, whereas pH focuses more on broader ecological and sociopolitical determinants, as a prior comparative analysis suggests [14]. Thus, we hypothesize a divergence in the scope of competencies between PHL and OHL, with PHL placing greater weight on interdisciplinary and systemic competencies, while OHL maintains a stronger connection to veterinary medicine.

### Expectation II) conceptual contrasts between EHL and PHL

Existing literature highlights the contrasts between EH, focusing on environmental hazards as direct human health risks, and PH, advocating for eco-centric interconnectedness from a systems-oriented perspective [18]. We anticipate that if the approaches PH and EH diverge in their theoretical foundations, their literacy concepts (EHL and PHL) will mirror these differences.

### Expectation III) inconsistent terminology in educational contexts

The preliminary literature search indicated a predominance of studies from the educational sector. While general *health education* often defines Health Literacy as its central goal and outcome [19], *health profession education* rarely incorporates literacy concepts in widely cited frameworks on sustainable healthcare and Planetary Health Education or distinguishes between literacy and competency [[20], [21], [22]]. We hypothesize a lack of differentiation between literacy and competency constructs in studies related to health profession education.

## Methods

We adopted content co-occurrence analysis (COA) to systematically quantify patterns and relationships between concepts and track their co-evolution over time [[23], [24], [25], [26]]. COA resulted in a cluster formation, which was supplemented by qualitative interpretation to contextualize quantitative findings and test the expectations noted in the introduction. This study is conducted according to recommendations of the manual for the bibliometric network viewer software VOSviewer [27] and follows the seven-step guidelines for bibliometric content analysis proposed by Klarin [28].

A predefined protocol was established, adhering to predefined inclusion/exclusion criteria and data analysis strategy aligned with Van Eck & Waltman and Klarin [27,28]. Yet, the formal registration in PROSPERO was not pursued since the scope of the study did not align with PROSPERO’s scope, but all information required to replicate this study is reported in the article and Supplementary Material.

### Co-occurrence analysis

COA examines the frequency and relational patterns of co-occurring terms within a dataset [[27], [28], [29]]. This allows the identification of patterns, clusters, and associations between concepts, providing insights into the intellectual structure of a research field [29,30]. The visualization map is formed by items (terms) and links, where each link represents a relation between a co-occurring set of terms. The strength of the links implies the frequency of co-occurring items in the corresponding publications. COA facilitates cluster formation by solving an optimization problem [31]. Clusters are groups of closely related items formed via VOS (‘visualization of similarities’) modularity-based techniques, which algorithmically group terms by co-occurrence strength and semantic proximity [26,28,29].

### Data collection strategy

The search strategy was collaboratively developed by all authors over a four-week period, utilizing an iterative process informed by a prior scoping review [17]. The strategy of this prior review was pilot-tested in the last week of January 2025 and was refined for this study’s research focus (no restrictions on population). We employed seed references, selected based on the identified literacy concepts in the prior scoping review, to benchmark our strategy. A librarian was consulted without refinement of our strategy.

The final search string (Supplementary material, file 1) comprised three thematic blocks: i) health; ii) literacy/competency; and iii) environment-related keywords and approaches. Eligibility criteria for screening (Supplementary material, file 2) were developed according to the PCC (population, concept, and context) framework [32] without restrictions on population or time. Peer-reviewed studies were included if they addressed literacy/competency concepts integrating health with ecological, climate, or sustainability dimensions. Studies are excluded if they fail to meet the interdependence of health and ecological determinants.

The search was conducted in the last week of February 2025. Web of Science was chosen to ensure VOSviewer-software compatibility and due to institutional availability aligning with recommendations [28]. The Web of Science 'All Data' search yielded 1599 records. For a complementary search strategy [28], two searchers JK and LR checked reference lists of indicative studies and Google Scholar (first 150 results screened for eligibility), identifying additional 81 studies.

**Fig. 1:**
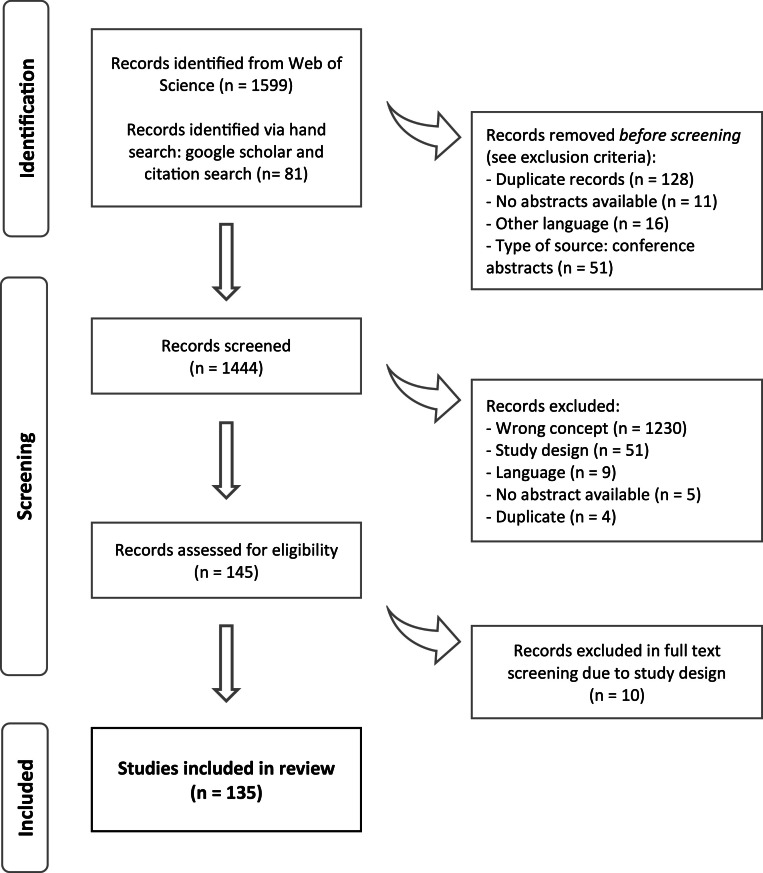
Data collection flow chart detailing the screening process for bibliometric content analysis.

We documented the screening process in a flow chart (Fig. 1). Following deduplication via reference management software Citavi, two researchers (LR/JK) independently screened the titles and abstracts (*n* = 1444), achieving 97% of inter-rater agreement [33] for the first 10% of records. Any uncertainties or discrepancies were resolved through team consensus. Following a full-text review (*n* = 145), 10 studies with non-compliant study design were excluded, resulting in a final set of 135 records. The keywords were manually appended to the dataset of titles and abstracts.

### Data analysis strategy

The dataset was first transformed for cluster formation into VOSviewer-software [27]. This software automatically excludes non-nouns/adjectives and calculates relevance scores for each item; a low relevance score suggests random co-occurrence patterns, typical of general terms, whereas a high relevance score indicates that a term consistently co-occurs with a specific set of terms, reflecting meaningful association [34]. In this study, terms with high relevance scores were classified as ‘Top Impact’. By default, the software selects the top 60% of terms by relevance score, and a minimum co-occurrence threshold of five mentions per term was chosen after previewing the data, as there are no clear recommendations for cut-offs, which depend on factors such as term count and desired figure visibility [27,28]. Excluded terms with three or four occurrences were extracted via VOSviewer’s text mining function and documented for transparency (Supplementary Material, file 3). As generally recommended, a thesaurus file [27] was applied to standardize synonyms, grammar and spelling, minimize redundancy in similar word groups, and remove overly generic terms [28,30] (Supplementary material, file 4). To analyze temporal patterns, an overlay visualization was used, where the color of the node (term) reflects the specific attribute (publication year) of the items [34].

The cluster formation of items was based on semantic proximity; however, study-to-cluster assignments were not apparent. Therefore, all 135 included studies were manually categorized in Excel by one reviewer (LR) and assigned to clusters according to the cluster items. The assignments were verified by a second reviewer (JK). After cluster-related studies were identified, study information was extracted using a predefined template. The data synthesis, as well as the labelling of each cluster, followed a qualitative interpretation of the studies' shared characteristics, emerging themes, and intersections within and across clusters.

## Results

The 135 included studies were predominantly conducted by researchers affiliated with institutions in the Global North (North America: 37%, Europe: 27%, Australia: 14%), while the representation of Asia (1 %), Africa (2%), and South America (4%) is limited. A breakdown of affiliations by country shows that low- and middle-income countries account for only 17% of first-authorships and 7% of co-authorships (Supplementary Material, file 5).

In VOSviewer, keywords with three or four co-occurrences, such as 'co-benefits', 'environmental footprint, 'mental health', and 'system thinking' (Supplementary material, file 3), are excluded despite their recognition in the broader research field due to the limited frequency of co-occurrences. Conversely, the terms with the highest number of co-occurrences (Supplementary material, file 6) are excluded due to low relevance scores, reflecting their weak conceptual associations and lack of selectivity within the network [26,29]. For example, while the terms ‘health literacy’ (28 co-occurrences) and ‘awareness’ (18) met the inclusion thresholds, others such as ‘education’ (75), ‘competency’ (60), ‘knowledge’ (56), and ‘skill’ (48) were omitted (Supplementary material, file 6).

**Fig. 2:**
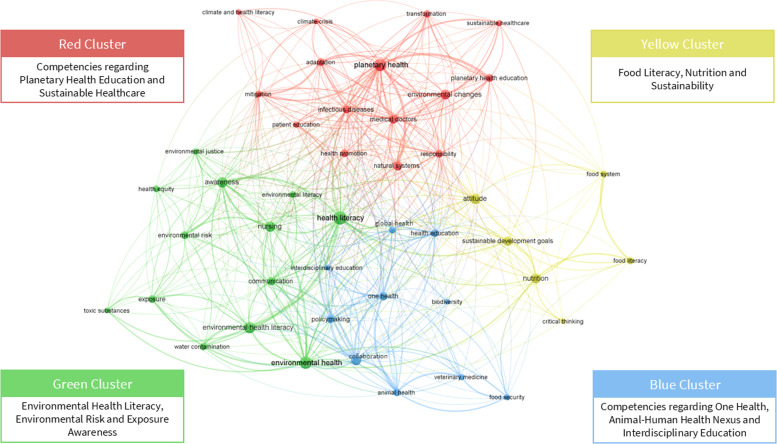
COA network and cluster visualization representing terms with at least five co-occurrences. Colored items and lines representing clusters. Line strength and node size indicative of higher numbers of connections and co-occurrences. Generated by VOSviewer software.

For terms exceeding the co-occurrence frequency threshold (≥5) and the relevance score cut-off (60%), the VOS-technique identified four distinct clusters, labelled for convenience by colours (red, green, blue, and yellow) as depicted in Table 1 and Fig. 2.

**Table 1:** Frequency and relevance score of co-occurring terms by cluster. *Top impact terms indicate the three items with the highest relevance score calculated via VOSviewer technique (approximated to two decimal places).

Cluster	Included terms (*frequency of co-occurrences*)	Top impact terms* (*relevance score*)	Indicative publications *(first author, year + title)*
*Red:* Competencies in Planetary Health Education and Sustainable Healthcare	Adaptation (9) Climate and Health Literacy (5) Climate Crisis (5) Environmental Changes (15) Health Promotion (9) Infectious Diseases (10) Medical Doctors (12) Mitigation (7) Natural Systems (14) Patient Education (6) Planetary Health (24) Planetary Health Education (10) Responsibility (9) Sustainable Healthcare (6) Transformation (8)	Sustainable Healthcare (2.02) Planetary Health Education (1.44) Transformation (1.35)	Bevan et al. (2023): Planetary health and sustainability teaching in UK medical education: A review of medical school curricula. Jacobsen et al. (2024): Planetary health learning objectives: foundational knowledge for global health education in an era of climate change. Limaye et al. (2020): Developing A Definition Of Climate And Health Literacy. Matlack et al. (2024): A scoping review of current climate change and vector-borne disease literacy and implications for public health interventions.
*Green*: Environmental Health Literacy and environmental risk and exposure awareness	Awareness (17) Communication (11) Environmental Health (23) Environmental Health Literacy (20) Environmental Justice (6) Environmental Literacy (7) Environmental Risk (10) Exposure (10) Health Equity (7) Health Literacy (28) Nursing (17) Toxic Substances (5) Water Contamination (8)	Toxic Substances (1.76) Exposure (1.33) Water Contamination (1.25)	Binder et al. (2022): Environmental Health Literacy as Knowing, Feeling, and Believing: Analyzing Linkages between Race, Ethnicity, and Socioeconomic Status and Willingness to Engage in Protective Behaviors against Health Threats. Fiore et al. (2024): Development and validation of the Environmental Health Literacy Index: a new tool to assess the environmental health literacy among university students. Gray et al. (2021): Knowledge and Beliefs Associated with Environmental Health Literacy: A Case Study Focused on Toxic Metals Contamination of Well Water. Lichtveld et al. (2019): Advancing Environmental Health Literacy: Validated Scales of General Environmental Health and Environmental Media-Specific Knowledge, Attitudes and Behaviors.
*Blue*: Competencies regarding One Health, animal-human health nexus and interdisciplinary education	Animal Health (9) Biodiversity (5) Collaboration (16) Food Security (6) Global Health (7) Health Education (10) Interdisciplinary Education (5) One Health (11) Policymaking (10) Veterinary Medicine (6)	Food Security (2.20) Veterinary Medicine (2.04) Animal Health (1.67)	Blankart et al. (2024): Health literacy, governance and systems leadership contribute to the implementation of the One Health approach: a virtuous circle. Estela-Galarza et al. (2022): Experiences of veterinary medicine intervention in the development of Healthy Municipalities in Latin America and in the Caribbean. Rabinowitz et al. (2017): Incorporating one health into medical education. Roopnarine et al. (2022): A Focus on Methodology: A Mixed-Methods Approach to Conduct a Comprehensive Evaluation of the Need for One Health Education for Medical and Veterinary Students in the Context of COVID-19.
*Yellow*: Food Literacy, nutrition, and sustainability	Attitude (16) Critical Thinking (5) Food Literacy (8) Food System (7) Nutrition (15) Sustainable Development Goals (14)	Food Literacy (3.26) Food System (2.93) Critical Thinking (2.11)	Kalkan et al. (2025). Are Nutrition Literacy and Sustainable Dietary Habits Associated with Cardiovascular Disease and Diabetes Developmental Risks? Mengi Çelik et al. (2025) Evaluation of the relationship between nutrition literacy, Mediterranean diet compliance, ecological footprint and sustainable environmental attitudes in adolescents. Park et al. (2020): Development of a Comprehensive Food Literacy Measurement Tool Integrating the Food System and Sustainability. Teng et al. (2022): Sustainable food literacy: A measure to promote sustainable diet practices.

### Red cluster: competencies in planetary health education and sustainable healthcare

The largest cluster (red cluster; 15 nodes) encompasses terms related to Planetary Health Education and sustainable healthcare (Table 1), highlighting 'transformation', 'adaptation', and 'climate crisis' as highly relevant topics.

While few studies focus on the general population [7,35], the majority of studies in the cluster target health professionals emphasizing a critical need for enhancing their competencies and literacy [6,8,[36], [37], [38], [39], [40], [41]]. A closer examination of the papers related to health profession education reveals two foci, with some studies promoting climate and health-related literacy [6,10,35,38] and others focusing on Planetary Health Education (learning objectives [36,22], knowledge and skills [8,39,41], transformative competencies [40], values and confidence [42]). A single review incorporates both a Planetary Health Education scope and a literacy concept, summarized as ‘eco-medical literacy’ without its further definition [43]. Others highlight its methodological relevance in instrumentation for PH training evaluation [41]. The integration of indigenous knowledge systems and their ecologically-comprehensive worldviews is also emphasized [44].

### Green cluster: environmental health literacy and environmental risk awareness

The green cluster (13 nodes) constitutes the second-largest thematic group, centring on EHL and environmental risk and exposure awareness. Highly scoring in relevance were the items ‘toxic substances’, ‘exposure’, and ‘water contamination’ (Table 1)

This cluster explores diverse EHL conceptualizations, from knowledge-focused terms like ‘knowledge and skills items’ [45], ‘environmental health risk knowledge’ [46], and ‘knowledge, attitudes, and behaviours’ [47] to distinctions such as Binder et al.’s ‘factual knowledge’ and ‘sufficiency knowledge’ (subjective preparedness) [48]. Broader frameworks categorize EHL as functional, interactive, and critical [49] while some adopt alternative terminology, such as ‘environmental cognition’ related to ‘risk perception and awareness’ [50]. Few studies explicitly link EHL to behavior, though Davis and colleagues advocate participatory approaches and contextual learning to bridge knowledge-behavior gaps [46], and others stress self-efficacy [46,48,51].

Measurement and instrument development dominate, with studies testing EHL in contexts such as: i) COVID-19 and industrial contaminants [48]; ii) rainwater harvesting/water contamination [46]; iii) rail-traffic/neighborhood noise [50]; iv) well contamination with toxic metals [51]; and v) air/food/water contamination [47]. Additionally, Fiore and colleagues validated a general EHL-index for university students [49]. These assessments of EHL did not refer to global themes such as climate change, biodiversity, or planetary boundaries.

### Blue cluster: competencies regarding one health, animal-human health nexus and interdisciplinary education

The blue cluster (10 nodes) focuses on competencies in the OH domain, with a particular emphasis on the highly-relevant keywords ‘food security’, ‘veterinary medicine’, and ‘animal health’ (Table 1). Foci in this cluster include interdisciplinary collaboration [[52], [53], [54]], systemic barriers [11,53,55,56], animal-human-health nexus [[52], [53], [54], [55], [56]], and climate change [52,54]. The majority of publications, including all educational studies, employ the term OH ‘*competencies’* [[52], [53], [54], [55], [56]], regarding for example zoonotic disease surveillance and outbreak response [53]. Our review contains a single study that explicitly defines and refers to OHL [11].

To reflect OH ‘competencies’, multiple studies stress updating curricula, with a notable focus on veterinary education [55,57], stating that veterinarians are the key OH actors [55]. However, some studies link the veterinary expertise to human health and policy, particularly in collaborative practice and interprofessional education, e.g. together with medical or Public Health education [[52], [53], [54]]. The *OHL* scope differs from the OH ‘*competencies’* scope in veterinary and educational settings: The key theme for OHL is governance and systems leadership, arguing that improved literacy drives policy advancements [11]. One study emphasizes the importance of competencies for leadership in municipal programs [56], but does not explicitly tie OH to governance or highlight the interdependence. Authors in the educational context focus on structural barriers in regard to enhancing OH ‘competencies’, including resource constraints, institutional silos and faculty expertise gaps [53,55].

### Yellow cluster: food literacy, nutrition, and sustainability

The yellow cluster (6 nodes) comprises the area of Food Literacy, nutrition, and sustainability with the key terms ‘food system’, ‘critical thinking’, ‘Sustainable Development Goals’, and ‘attitude’ (Table 1).

This cluster examines Food Literacy’s role in ecological sustainability, exploring how informed dietary choices influence individual health and environmental outcomes [[58], [59], [60], [61], [62]]. Studies analyze concepts like Health Literacy [62], ‘nutrition literacy’ [63,64] or ‘Food Literacy’ [[59], [60], [61]] spanning individual behaviors (take-out consumption [64], vegan diets [59]) and systemic factors (food safety, production, policy) [58,60,61]. A ‘comprehensive Food Literacy’ framework extends traditional literacy dimensions (functional, interactive, critical) to food system stages (production to disposal), emphasizing systemic and relational competencies [61]. Similarly, the systemic competencies in individuals’ food literacy are highlighted [59]. The cluster’s studies target school education and children [58], adolescents [64], consumers [59,62], general population [60,61], and patients’ health outcomes [63], while notably omitting health professionals. The approaches EH, OH, PH are not mentioned.

### Single item patterns and temporal development

**Fig. 3:**
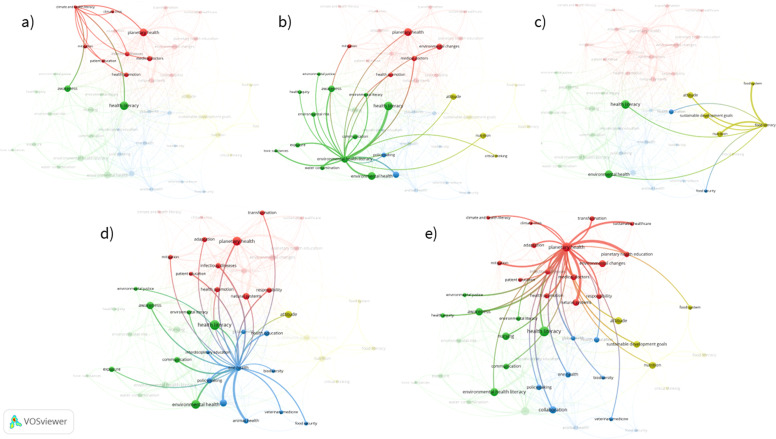
Network patterns of selected key items and concepts. Five terms indicative of key concepts analyzed individually. a) Climate (Change) and Health Literacy; b) Environmental Health Literacy; c) Food Literacy; d) One Health; e) Planetary Health. The item ‘Climate and Health Literacy’ exhibits conceptual overlaps with PH mainly within the red cluster (Fig. 3a). The item ‘Environmental Health Literacy’ demonstrates the strongest connections within its own cluster, as indicated in quantity and in line strength, with weaker connections to ‘critical thinking’, ‘nutrition’, ‘Planetary Health’, and ‘mitigation’ (Fig. 3b). The item ‘Food Literacy’ is characterized by its connections with food-related items and a weak link to Health Literacy (Fig. 3c). ‘One Health’ shows multiple connections to other clusters but lacks direct connections to ‘Food Literacy’ in the yellow cluster (Fig. 3d). The item ‘Planetary Health’ exhibits a broad field with multiple connections to all clusters (Fig. 3e).

For analyzing the item pattern of specific terms indicative of key concepts in VOSviewer [27] only their connections are visualized, and others are pushed into the background (Fig. 3).

**Fig. 4:**
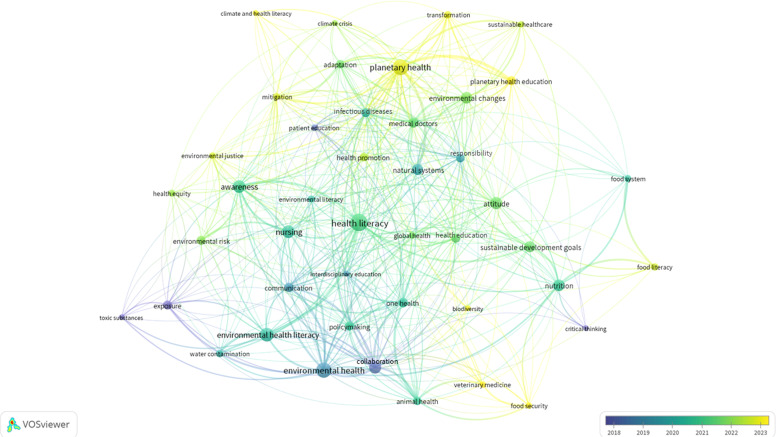
Temporal development network visualization with colors indicating publication year. Oldest publication from 1996 and latest publication from 2025. Co-occurring items in older publications indicated by darker colors in the network visualization. The darker colors in the network visualization indicate the presence of co-occurring items in the older publications (2018 and before). The items (‘exposure’, ‘toxic substances’, ‘patient education’, ‘Environmental Health’) demonstrate the first research interest in understanding environmental health risks and exposure. In the publications from 2019–2020, the terms ‘Environmental Health Literacy’, ‘One Health’, and ‘infectious diseases’ became prominent co-occurring items, reflecting a broader understanding of environmental determinants of health. The studies from 2021–2022 mark a greater emphasis on ‘adaptation’ and ‘transformation’ in the health competency and literacy domain. The most recent studies (2023 and later) have brought up new items: ‘Planetary Health Education’, ‘environmental justice’, ‘mitigation’, ‘climate and health literacy’, ‘biodiversity’, ‘Food Literacy’, and ‘food security’, indicating an expansion of literacy and competency focusing on the interconnectedness of human, animal, and ecological health.

The keywords and main themes of literacy concepts have developed over the past decade. Fig. 4 visualizes how this transitioning process is reflected in the co-occurring items and themes. The earliest included publication dates back to 1996 and investigated EHL; the full overview about the study distribution over time is available in Supplementary material file 7.

## Discussion

The COA results yielded four distinct clusters, indicative of research trajectories and priorities. The *red cluster* centered on Planetary Health Education, climate-ecosystem interdependencies, transformative competencies, and sustainable healthcare. The *green cluster* prioritized EHL regarding specific environmental health risks and methodological or equity-focused advancements, with less attention to ecological determinants. The *blue cluster* highlighted interdisciplinary collaboration, systemic barriers, and the animal-human-health nexus, with a priority of OHL on systems and governance. The *yellow cluster* is uniquely centered on Food Literacy’s role in linking nutrition, health, and sustainability, not mentioning EH, OH, or PH. The single item pattern analysis underscored OH and PH as broadly integrative themes, whereas EHL, ‘Climate and Health Literacy’, and ‘Food Literacy’ remained cluster-specific (Fig. 3).

### Discussion of the results in light of prior expectations

#### Differentiation of concepts and scopes between OHL and PHL

The first of prior expectations was that literacy concepts (OHL vs. PHL) will align with the distinct scopes of their parent approaches, with OHL emphasizing human–animal interaction and PHL prioritizing systems-orientation.

While the red and blue clusters reflect themes related to OHL and PHL, few of included studies explicitly address and define these concepts [9,11]. OHL and PHL have similar scopes in their definitions, building on Sørensen’s widespread Health Literacy concept [65]. Item pattern and temporal analyses (Figs. 3, 4) reveal conceptual similarities between the two clusters, emphasizing that both are emerging research fields and build comprehensive, integrative concepts to address health within anthropogenic climate impacts and biospheric interactions with systemic and equity-centered solutions.

Climate-specific Health Literacy aligns conceptually with PH, as it prioritizes climate crises but also underscores broader ecosystem interdependencies [6,7]. Similarly, climate change is of key interest in the OH cluster [52,54]. ‘Biodiversity’ resides in the blue OH cluster, though also PH-relevant [66]. The findings align with the OH definition’s conceptual expansion in 2021 as “an integrated, unifying approach that aims to sustainably balance and optimize the health of people, animals, and ecosystems” [67]. Correspondingly argue Amuasi and Winkler that “a view that One Health is focused on human–animal interaction alone, primarily addresses the risk of zoonotic events […] is flawed” [15].

However, Castañeda and colleagues highlight a persistent focus of OH research on zoonoses and infectious diseases [14], contrasting with our results, which associate ‘infectious diseases’ with the red cluster. Additionally, our results state the focus on human-animal health interactions merely for OH in educational settings and not for OHL’s conceptual underpinning: while OHL emphasizes governance and systems leadership [11], OH competencies remain rooted in veterinary education [[52], [53], [54], [55], [56], [57]]. This suggests a distinction between OHL and OH competencies when reflecting the prior expectation.

Despite historical ties that associate ‘climate and health’ with PH/PHL and OH competencies with veterinary fields, we reject our initial expectation regarding the conceptual foundation of OHL and PHL, both being comprehensive and integrative approaches, as revealed by analyses of key words (Table 1) and item patterns (Fig. 3) and corroborate recent developments of a shared Planetary and One Health Literacy vision [68].

#### Differentiation of concepts and scopes between EHL and PHL

Second, we expected EHL and PHL to reflect differing conceptual priorities, with EHL emphasizing human-centric hazards and PHL prioritizing eco-centric systems thinking.

Our analysis shows EHL as the comparatively oldest research domain (Fig. 4), and with 20 co-occurrences (Table 1) the most established of included concepts. In contrast, PHL emerged as a nascent conceptual framework, forming a distinct research trajectory (Fig. 4). This aligns with prior observations stating exponential growth in PH-related publications after 2017, reflecting heightened interdisciplinary interest [18].

Structurally, PHL derives its conceptual framework from established Health Literacy dimensions by Sørensen and colleagues [9,65], whereas studies employing EHL as ‘knowledge and skills items’ [45] or ‘environmental health risk knowledge‘ [46] rather than adopting Health Literacy’s structured domains (access, understand, appraise, apply) [65].

Thematically, studies explicitly examining EHL address contexts spanning from well water contamination [46,51], industrial pollutants [48], air pollution [47] to traffic noise [50], demonstrating its focus on localized and proximate hazards and individual risk mitigation. Additionally, substantive differences in key items (Table 1) and in single-item patterns (Fig. 4) corroborated our expectation that EHL misses non-anthropocentric considerations and predominantly addresses micro- and meso‑level determinants, whereas PHL adopts a holistic systems perspective encompassing all ecological and societal determinants. This aligns with the rejection of anthropocentric worldviews at a PH conceptual level [3,69].

Collectively, the findings confirm prior expectations of conceptual distinctions between EHL and PHL.

#### Inconsistent terminology in educational contexts

Third, it was anticipated that studies with an educational scope would lack clarity between literacy constructs and discrete competency sets.

Multiple studies in the PH- and OH-related clusters focused on the topics of health profession education and curriculum development. This emphasis aligns with the arguments presented by Jochem and colleagues that comprehensive literacy most effectively equips health professionals to address ecological challenges while advancing human health [70]. Recent literature further underscores literacy’s critical role when emphasizing that health professionals require competencies extending beyond knowledge acquisition, including decision-making and ethical responsibility [40,54,71]. However, other recommendations extend beyond health profession education, emphasizing the importance of broaden scope addressing both formal and non-formal education of civil society [13]. To design effective interventions for both health professions’ curricula and non-formal educational contexts, measurement and assessment tools based on theory-driven and standardized approaches are required [72].

Our quantitative analysis showed frequent use of terms like ‘competency’ (60 co-occurrences) ‘knowledge’ (56), and ‘skill’ (48), indicating greater use compared to precise literacy concepts, for example ‘health literacy’ (28). However, these broad terms had low calculated relevance scores, reflecting their weak cluster selectivity. This suggests a generality of the terms with further need for differentiation and specification when using.

The heterogeneity and fragmentation in the Planetary Health Education field is confirmed by Carrion and colleagues [73]. Similarly, our findings regarding studies in the cluster of health profession education and Planetary Health Education reveal inconsistent use of terminology regarding *skills, learning objectives*, and *competencies*, despite the studies’ considerable contributions to curriculum design and transformation of educational paradigms [36,[39], [40], [41],^22^,^42^,^43^].

### Research gaps

Further research should disaggregate the dominance of Global North to advance the field by incorporating diverse perspectives. Additionally, the emerging overlaps between OHL and PHL suggest potential for integration. Further research should clarify distinctions or develop integrated models (e.g., *Planetary and One Health Literacy)*. Our analyses revealed that current literature lacks explicit differentiation between competency and literacy conceptualization, particularly in OH education/OHL as well as PH education/PHL. Future work must understand and differentiate these constructs to replace ambiguous terminology. Specifically, further research is needed to differentiate between *literacy, learning objectives* and *competency* requirements used in educational frameworks.

### Limitations

Our study has several limitations. First, there is overall little published literature explicitly addressing comprehensive literacy concepts. Thereby, not all concepts are displayed within the quantitative built clusters, potentially overlooking emerging frameworks (e.g., Ecohealth literacy). Second, COA relied on title/abstract data, omitting conceptual explanations if only stated in full-texts. Third, manual thesaurus filtering may introduce observer bias, although it is recommended to enhance clarity in cluster building and visualization. Fourth, a significant limitation of bibliometric analyses in general is the absence of consensus-based reporting standards. Despite these constraints, our findings provide foundational insights initiating a theoretical research trajectory.

## Conclusions

Despite methodological and conceptual diversity, the results consistently highlight the importance of fostering literacy/competencies that reflect the reciprocal relationship between humans and natural systems. The anticipated conceptual distinction between OHL and PHL could not be confirmed. In contrast, a clearer difference emerged between EHL and PHL: while EH reflects an anthropocentric orientation that is reflected by the EHL concept, PHL incorporates planetary and systemic interdependencies as well as non-human wellbeing. The analyses also reveal widespread use of vague terms (e.g., knowledge, attitude, skill) over precise literacy constructs, with these terms lacking contextual specificity and scoring low in relevance. These findings emphasize the need for standardized terminology to operationalize literacy/competency concepts for further research, particularly in instrument development or educational research.

## Declaration of generative AI in scientific writing

During the preparation of the manuscript, the authors used Deep Seek R1 (academiccloud.de) for scientific English optimization and spell-checking. After using this tool, the authors reviewed and edited the content as needed and take full responsibility for the content of the published article.

## Funding

This research did not receive any specific grant from funding agencies in the public, commercial, or not-for-profit sectors.

## Data availability

The original contributions presented in the study are included in the article/supplementary material, further inquiries can be directed to the corresponding author.

## CRediT authorship contribution statement

**Lydia Reismann:** Writing – review & editing, Writing – original draft, Methodology, Formal analysis, Data curation, Conceptualization. **Jennifer Kallenbach:** Writing – review & editing, Formal analysis, Data curation. **Carmen Jochem:** Writing – review & editing, Supervision, Methodology, Formal analysis, Data curation.

## Declaration of competing interest

The authors declare that they have no known competing financial interests or personal relationships that could have appeared to influence the work reported in this paper.

## Appendix Supplementary materials

Image, application 1

Image, application 2

Image, application 3

Image, application 4

Image, application 5

Image, application 6

Image, application 7

## Footnotes

Supplementary material associated with this article can be found, in the online version, at doi:10.1016/j.joclim.2026.100700.
