# Supplementary material for: Understanding health literacy within the nexus of environmental, planetary, and one health: Mapping the evidence via bibliometric content analysis
Source: J Clim Chang Health. 2026 Jun 26;30:100700. doi: 10.1016/j.joclim.2026.100700 (PMC13319369; doi:10.1016/j.joclim.2026.100700)
Supplement: Supplementary file 1 [file mmc1.docx]

##### Additional file 1: Structural Decomposition of Search Terms for ‘Web of Science’

|  | *Related search terms* |
| --- | --- |
| **Population/setting** | Health AND |
| **Concept** | ("Health Literacy" OR literacy* OR competenc*) NEAR/9 (model OR theory OR framework OR approach OR concept OR strategy OR defin* OR understanding OR characteris* OR identif* OR propos*) AND |
| **Context** | "Education for Sustainable Development" OR "Environmental Health" OR "Environmental Sustainability" OR "Conservation of Natural Resources" OR "Carbon Footprint" OR "Climate Change" OR eco$health* OR (eco* NEAR/1 health*) OR one$health OR (one NEAR/1 health) OR ((medic* OR health*) NEAR/3 (planet* OR biodiversity* OR ecological)) OR (global* NEAR/1 warm*) OR (climate* NEAR/3 (act* OR chang* OR resilien* OR protect* OR mitigat* OR adaptation* OR crisis OR distress*)) OR climate*sensitive OR pro*environmental OR (sustainab* NEAR/3 (resilien* OR environmental OR development* OR ecolog* OR transform* OR educat*)) |
