# Supplementary material for: Understanding health literacy within the nexus of environmental, planetary, and one health: Mapping the evidence via bibliometric content analysis
Source: J Clim Chang Health. 2026 Jun 26;30:100700. doi: 10.1016/j.joclim.2026.100700 (PMC13319369; doi:10.1016/j.joclim.2026.100700)
Supplement: Supplementary file 2 [file mmc2.docx]

##### Additional file 2: Inclusion and exclusion criteria for bibliometric content analysis

|  | **Inclusion criteria** | **Exclusion criteria** |
| --- | --- | --- |
| **Population** | This review does not have any restrictions on population. | |
| **Concept** | Articles considering theoretical foundation, models or concepts of competencies or literacy. | Articles without conceptual foundation of competencies or literacy. |
| **Context** | Competencies or literacy concepts integrating health and planetary, climate, environmental, or sustainability aspects. | Solely addressing health literacy without planetary, climate, or environmental aspects.  OR  Solely addressing environmental related competencies or literacy concepts without health aspects. |
| **Time** | Published from inception to February 28, 2025 | Publications from March 1, 2025, onwards |
| **Type of sources** | Primary research  Secondary research | Media  Blogs  Editorials  Commentaries  Thesis and dissertations  Conference abstracts |
| **Language** | Title and abstract available in English | All other languages |
