# Supplementary material for: Understanding health literacy within the nexus of environmental, planetary, and one health: Mapping the evidence via bibliometric content analysis
Source: J Clim Chang Health. 2026 Jun 26;30:100700. doi: 10.1016/j.joclim.2026.100700 (PMC13319369; doi:10.1016/j.joclim.2026.100700)
Supplement: Supplementary file 3 [file mmc3.docx]

##### Additional file 3: Examples of excluded keywords with 3 or 4 co-occurences

Exclusion based on number of co-occurences below the threshold of 5 co-occurences

| **Term** | **Co-occurrences** |
| --- | --- |
| co-benefits | 4 |
| environmental footprint | 4 |
| mental health | 3 |
| resilience | 4 |
| sustainable food literacy | 3 |
| system thinking | 3 |
