# Supplementary material for: Understanding health literacy within the nexus of environmental, planetary, and one health: Mapping the evidence via bibliometric content analysis
Source: J Clim Chang Health. 2026 Jun 26;30:100700. doi: 10.1016/j.joclim.2026.100700 (PMC13319369; doi:10.1016/j.joclim.2026.100700)
Supplement: Supplementary file 4 [file mmc4.docx]

##### Additional file 6: Excluded, highly co-occurring items (≥10) sorted by number of co-occurrences.

| term (≥ 10 co-occurences) | Number of co-occurences | Excluded by relevance score ≤ 60%* |
| --- | --- | --- |
| education | 75 | excluded |
| competency | 60 | excluded |
| knowledge | 56 | excluded |
| climate change | 53 | excluded |
| skill | 48 | excluded |
| healthcare professionals | 41 | excluded |
| public health | 31 | excluded |
| sustainability | 29 | excluded |
| health profession education | 27 | excluded |
| leadership | 14 | excluded |

*Automatically excluded by VOSviewer algorithms
